# Maternal Dietary Anthocyanidin, Dietary Inflammatory Potential, and Risk of Small-for-Gestational-Age in China

**DOI:** 10.3390/nu17203187

**Published:** 2025-10-10

**Authors:** Binyan Zhang, Kun Xu, Baibing Mi, Hong Yan, Duolao Wang, Shaonong Dang, Ke Men

**Affiliations:** 1 School of Public Health, Xi’an Medical University, Xi’an 710021, China; zhangbinyan@stu.xjtu.edu.cn; 2 Department of Epidemiology and Biostatistics, School of Public Health, Xi’an Jiaotong University Health Science Center, Xi’an 710061, China; xukun_728@hust.edu.cn (K.X.); xjtu.mi@xjtu.edu.cn (B.M.); yanhonge@mail.xjtu.edu.cn (H.Y.); 3 Department of Nutrition and Food Hygiene, School of Public Health, Tongji Medical College, Huazhong University of Science and Technology, Wuhan 430030, China; 4 Department of Clinical Sciences, Liverpool School of Tropical Medicine, Liverpool L7 8XZ, UK; duolao.wang@liverpool.ac.uk

**Keywords:** pregnancy, anthocyanidin, dietary inflammatory index, small-for-gestational-age

## Abstract

**Background:** The interaction between anthocyanidin intake and dietary inflammatory potential might influence small-for-gestational-age (SGA), but the available evidence has been limited. This study aims to examine the associations of anthocyanidin with SGA and whether these associations change with dietary inflammatory potential. **Methods:** Data were derived from 2244 pregnant women enrolled in a community-based, randomized controlled trial between 2015 and 2019 in China. Anthocyanidin intake was calculated with the use of validated food-frequency questionnaires. The energy-adjusted dietary inflammatory index (EDII) was determined by aggregating data from 33 food parameters. Infant birth outcome measurements were obtained from hospital records. Associations were assessed by generalized estimating equations with adjustment for confounding factors. **Results:** During 39.7 gestational weeks of follow-up, 234 SGA cases occurred. The median intake of anthocyanidin was 28.7 mg/d. Higher consumption of total anthocyanidins (OR: 0.96, 95% CI: 0.95 to 0.97), cyanidin (OR: 0.96, 95% CI: 0.95 to 0.97), and peonidin (OR: 0.96, 95% CI: 0.96 to 0.97) subclasses was associated with a reduced risk of SGA. EDII was associated with an increased risk of SGA (OR: 1.08, 95% CI: 1.03 to 1.12). In addition, we observed that higher anthocyanidin intake was inversely associated with EDII (β: −0.40, 95% CI: −0.46 to −0.34). The inverse anthocyanidin-SGA association was mostly greater among women in the highest tertile of EDII (OR: 0.67, 95% CI: 0.65 to 0.68) compared with the lowest tertile. **Conclusions:** Higher anthocyanidin intake was inversely associated with SGA, especially among women with higher EDII scores.

## 1. Introduction

The phenotypic small-for-gestational-age (SGA) was defined as failure to achieve its genetic growth potential based on international standards [1,2]. It remained a critical global health issue, with an estimated 23.3 million infants born SGA in 2012 and 23.4 million in 2020 [3,4], accounting for 22.4% of the 2.4 million annual neonatal deaths [4]. Beyond mortality, SGA survivors were at increased risk for lifetime health and developmental problems, leading to loss of human capital and vulnerable societies [5,6]. Evidence-based antenatal interventions to reduce the incidence of SGA, such as multiple micronutrient and protein-energy supplements, had been proven effective [7]. However, WHO recommendations regarding these interventions were restricted to undernourished populations [7,8]. To date, timely termination of pregnancy was the definitive intervention but carried an increased risk of preterm birth, highlighting the urgent need for more effective and safer interventions.

As a simple and practicable non-pharmacological intervention, dietary modification has been well-established to have many health benefits [9,10]. An adequate maternal diet during pregnancy might be an important modifiable risk factor for SGA prevention [10,11]. Evidence indicated that higher consumption of vegetables, fruits, and whole grains was associated with a reduced risk of SGA [11,12,13], yet the underlying bioactive compounds had not been fully elucidated [14,15,16]. An anti-inflammatory state of pregnancy was acknowledged to be associated with fetal growth [17]. The potential role of diet in preventing SGA might be partly attributed to the intake of dietary phytochemicals with anti-inflammatory properties, such as anthocyanidins [18,19,20].

Anthocyanidins, a member of the flavonoid class of secondary metabolite, were water-soluble plant pigments. Structurally, they were flavylium cations, characterized by a C_6_-C_3_-C_6_ carbon skeleton. Owing to their poor stability, anthocyanidins most commonly occurred in nature in their glycosylated forms, known as anthocyanins [21]. More than 600 anthocyanins derived from anthocyanidins have been identified in nature [22]. Among these anthocyanidins, delphinidin, cyanidin, and peonidin occurred ubiquitously and had dietary importance. These compounds were commonly consumed in the human diet, especially in red, blue, or purple fruits and vegetables, as well as in certain cereals [23]. A randomized, double-blind, placebo-controlled trial further confirmed that anthocyanin supplementation improved anti-inflammatory capacity [24]. Epidemiological evidence suggested that maternal consumption of a more pro-inflammatory diet during pregnancy had been associated with an increased risk of SGA births [25], suggesting that dietary inflammatory potential might have an important moderating role on the anthocyanin-SGA associations. However, it remained unclear whether dietary anthocyanidins could protect against SGA and to what extent this association was modified by dietary inflammatory potential.

We hypothesized that anthocyanidin intake would protect against SGA births and that women who consumed higher levels of a pro-inflammatory diet during pregnancy would benefit more. The current study aimed to investigate the associations of maternal dietary anthocyanidin and its subclasses with SGA and to examine the extent to which dietary inflammatory potential may modify these associations in a cohort of pregnant women in China.

## 2. Materials and Methods

### 2.1. Study Population

This prospective population-based study analyzed data from a community-based, randomized controlled trial (RCT) assessing maternal multivitamin supplementation effects on birth outcomes (ClinicalTrials.gov NCT02537392). The trial methodology had been previously described in detail [26]. In brief, this community-based intervention was conducted across three rural counties (Bin, Xunyi, and Changwu) in western China from July 2015 to December 2019. Eligible participants were pregnant women aged 15–47 years with a gestational age of <20 weeks at enrollment. Participants were randomly allocated at the township level in a 1:1:1 ratio to receive either 400 μg folic acid alone, 400 μg folic acid plus 60 mg iron, or 400 μg folic acid combined with a B-complex vitamin containing 2 mg vitamin B_1_ (thiamine), 2 mg vitamin B_2_ (riboflavin), 2 mg vitamin B_6_ (pyridoxine), 2 μg vitamin B_12_ (cobalamin), 5 mg calcium pantothenate, and 15 mg nicotinamide. The present analysis initially included 2438 eligible participants with complete dietary data. Based on predefined exclusion criteria, we subsequently excluded 70 participants lost to follow-up, 16 who voluntarily withdrew, 20 cases of fetal loss, 4 stillbirths, 24 with hypertension or diabetes, 17 with missing birthweight data, and 43 reporting implausible energy intake (<500 or >5000 kcal/day), yielding a final analytical sample of 2244 participants (Figure S1).

Written informed consent was obtained from all participants, and ethical approval for the study was obtained from the Ethics Review Committee of Xi’an Jiaotong University Health Science Center (No. 20120008, approved on 6 March 2012).

### 2.2. Assessment of Dietary Anthocyanidin

Maternal dietary intake during pregnancy was assessed through face-to-face interviews using a validated 107-item Food Frequency Questionnaire (FFQ) during follow-up visits after enrollment [27,28]. The FFQ was designed to capture foods commonly consumed by rural populations in western China. Specifically, participants were asked to report both the frequency (8-tier frequency scale ranging from “almost never’ to “≥2 times/day”) and portion sizes of each food item during the most recent pregnancy. Based on these responses, the average daily intake for each food item was calculated.

The estimation of dietary anthocyanidin intake and its subclasses of delphinidin, cyanidin, and peonidin was based on FFQ data by referencing the China Food Composition Table (Standard Edition) [29]. This database included anthocyanidin content for 346 food items. We could not assume that foods that were not in the reference database did not contain anthocyanidins. Therefore, anthocyanidin intake estimation accounted for database limitations by incorporating three data treatment strategies. First, we assigned logical zeros (71.0% of items in FFQ) to animal products and non-pigmented plants where anthocyanidins were biologically absent. Second, we estimated values (12.1%) through nutritional equivalency modeling from analogous foods in both the FFQ and the reference database. Therefore, 83.2% of food items were assigned estimated values. Only processed foods (16.8%) without reference data received conservative zero assignments. The intake of anthocyanidin subclasses was calculated as Σ[consumed amount (g/day) × anthocyanidin content (mg/100 g)] for all foods. Total anthocyanidin intake was calculated by summing the intake of anthocyanidin subclasses. To analyze the association between anthocyanidins and SGA, we classified individuals based on quartile cut-off points of anthocyanidin intake.

### 2.3. Assessment of Dietary Inflammatory Index

The Dietary Inflammatory Index (DII) was developed to evaluate an individual’s dietary inflammatory potential [30]. Briefly, the DII was derived from a systematic review of 1943 articles (1950–2010) to account for the effects of 45 food parameters on six inflammatory biomarkers (IL-1β, IL-4, IL-6, IL-10, TNF-α, and CRP) that were upregulated in non-specific immune responses. In our analysis, 33 of the original 45 food parameters were available to calculate the DII. Pro-inflammatory parameters included energy, carbohydrates, cholesterol, protein, total fat, saturated fat, vitamin B_12_, and iron, and anti-inflammatory parameters included monounsaturated fatty acids (MUFA), polyunsaturated fatty acids (PUFA), *n*-3 fatty acids, *n*-6 fatty acids, fiber, vitamin A, β-carotene, thiamin, riboflavin, niacin, vitamin B_6_, folic acid, vitamin C, vitamin D, vitamin E, magnesium, zinc, selenium, caffeine, alcohol, isoflavones, anthocyanidins, green/black tea, onion, and garlic. To calculate the DII score, the pregnant women’s daily intake of each food parameter was transformed into z-scores by subtracting the global daily mean intake and dividing by the standard deviation. These z-scores were then converted to a percentile score to minimize the effect of right skewing. Each percentile value was subsequently doubled and centered by subtracting 1. The centered values were then multiplied by their corresponding inflammatory effect scores and aggregated to generate the composite DII score. Finally, the energy-adjusted DII (EDII) was derived using the residual method [31]. To explore the association between anthocyanidins and SGA at different degrees of EDII, we classified individuals based on tertile cut-off points of EDII.

### 2.4. Assessment of Small-for-Gestational-Age

Infant birth outcome measurements, including birth weight and gestational age at delivery, were obtained from hospital records. Gestational age- and sex-specific birthweight z-scores were derived using the INTERGROWTH-21st standards [1], and neonates with z-scores below the 10th percentile were classified as SGAs.

### 2.5. Assessment of Covariates

According to expert consensus regarding the pathogenic mechanisms of SGA and previous epidemiological studies [10,32,33], we identified and collected a set of potential covariates. These included maternal age, socioeconomic status, season of birth, parity, passive smoking, multivitamin supplements, maternal body mass index (BMI) at enrollment, pregnancy-induced hypertension or gestational diabetes mellitus, and total energy intake. Maternal age was analyzed as a continuous variable. Socioeconomic status was derived via principal component analysis of five indicators, including household wealth index, parental education, and parental occupation, and categorized into tertiles (lower, medium, and upper). Season of birth was classified as spring, summer, autumn, and winter. Parity was dichotomized into nulliparous or multiparous. Passive smoking was defined as exposure to secondhand smoke for more than 15 min daily during pregnancy. Multivitamin supplements were classified as folic acid alone, folic acid plus iron, or folic acid with B-complex vitamins. Maternal BMI at enrollment (kg/m^2^) was derived by dividing weight (kg) by height squared (m^2^).

### 2.6. Statistical Analyses

We conducted statistical analyses using SAS version 9.4 (SAS Institute, Cary, NC, USA). Descriptive analyses were performed to characterize the study population and food parameters across quartiles of anthocyanidin intake (Q1–Q4), presenting continuous variables as either median (interquartile range, IQR) or mean ± standard deviation (SD) depending on distribution normality or not, and categorical variables as frequencies (percentages). We employed generalized estimating equations (GEE) with adjustment for country-level clustering to assess differences across quartiles of total anthocyanidin intake [34]. Anthocyanidin and food parameters were standardized to mitigate skewness. Pearson correlation analysis was used to assess the associations between total anthocyanidin intake and food parameters. To assess the relative contribution of different dietary sources to variation in anthocyanidin intake among pregnant women, forward linear regression was employed. GEE was used to assess the odds ratios (ORs) with 95% confidence intervals (95%CIs) for the associations of dietary anthocyanidin intake and EDII with the risk of SGA. To explore the relationships of dietary anthocyanidin intake with EDII, GEE was also used to estimate the coefficient (β) and 95%CI. For all analyses, we modeled anthocyanidin intake and EDII both as continuous variables and in quartiles. We developed two hierarchical regression models to account for potential confounding factors. Model 1 adjusted for country-level clustering, while Model 2 was additionally adjusted for maternal age, socioeconomic status, season of birth, parity, passive smoking, multivitamin supplements, maternal BMI at enrollment, pregnancy-induced hypertension or gestational diabetes mellitus, and total energy intake. To assess potential effect modification by dietary inflammatory potential, interactions between the dietary anthocyanidin intake and EDII on SGA were evaluated by adding the interaction term (anthocyanidins × EDII) to the final adjusted model. Accordingly, we further performed stratified analyses by the tertiles of EDII to evaluate how anthocyanidin–SGA associations varied across EDII levels. A two-sided *p* value < 0.05 was considered statistically significant.

## 3. Results

### 3.1. Population Characteristics

During 39.7 gestational weeks of follow-up among the 2244 pregnant women, we documented 234 incident cases of SGA. Baseline characteristics of the participants according to quartiles of anthocyanidin intake were shown in Table 1. Dietary assessment was conducted at a mean gestational age of 26.2 ± 12.4 weeks. The median daily intake of anthocyanidin was 28.7 mg, with an intake range of 0.0–990.7 mg/d. Compared to women in the lowest quartile of anthocyanidin intake, those in the highest quartile were more likely to deliver in autumn, were multiparous, and had a lower incidence of pregnancy complications and SGA, as well as lower EDII scores (*p* < 0.05). The distributions of multivitamin supplements, maternal age, socioeconomic status, BMI, passive smoking, birth weight, and gestational age were similar across quartiles of anthocyanidin intake (*p* > 0.05). Anthocyanidin intake was most strongly correlated with the intake of fiber, vitamin C, and vitamin B_6_ (*r* ranging from 0.31 to 0.35, *p* < 0.001). However, no significant association was observed between anthocyanidin intake and the intake of caffeine, alcohol, green/black tea, or garlic (*p* > 0.05) (Table 2).

### 3.2. Anthocyanidin and Its Food Source

Table 3 presented the primary dietary contributors to total anthocyanidin intake and its subclasses. Watermelon accounted for the majority (98.6%) of the variation in total anthocyanidin intake among pregnant women, followed by sweet potato (0.6%) and grapes (0.4%). For delphinidin intake, the top three contributing foods were eggplant (49.5%), sweet potato (30.7%), and grapes (16.6%). In the case of cyanidin, watermelon was the predominant source, explaining 99.5% of the observed variation, with minimal contributions from plum/apricot (0.3%) and eggplant (0.1%). Regarding peonidin, sweet potatoes and grapes were the primary sources, accounting for 58.4% and 41.7% of the variation, respectively.

### 3.3. Anthocyanidin, Dietary Inflammatory Potential, and Small-for-Gestational-Age

The median daily intakes of total anthocyanidin and its subclasses were as follows: 28.67 mg for total anthocyanidins (IQR: 13.29–67.78 mg), 0.83 mg for delphinidin (IQR: 0.44–1.69 mg), 23.56 mg for cyanidin (IQR: 8.40–62.56 mg), and 2.20 mg for peonidin (IQR: 0.88–4.64 mg). The mean EDII score was -0.002 (SD 1.805). Our multivariable-adjusted analysis demonstrated inverse associations between dietary anthocyanidin intake and SGA risk, with a consistent 4% risk reduction per SD increase for total anthocyanidins (OR: 0.96, 95% CI: 0.95 to 0.97), cyanidin (OR: 0.96, 95% CI: 0.95 to 0.97), and peonidin (OR: 0.96, 95% CI: 0.96 to 0.97). However, delphinidin intake showed no significant association (OR: 1.03, 95% CI: 0.99 to 1.07) (Table 4).

In multivariable-adjusted models, participants in the highest quartile of total anthocyanidin intake, with a median of 131.88 mg/d, had a 19% lower risk of SGA (OR: 0.81, 95% CI: 0.66 to 0.98) compared to those in the lowest quartile. Similarly, participants in the highest quartile of cyanidin intake, with a median daily consumption of 125.08 mg/d, had a 17% lower risk of SGA (OR: 0.83, 95% CI: 0.71 to 0.97) compared to the lowest quartile. Those in the highest quartile of peonidin intake, who consumed a median of 9.85 mg/d, demonstrated an 11% risk reduction in SGA (OR: 0.89, 95% CI: 0.79 to 1.00). Conversely, a positive association was observed between EDII and SGA risk (OR: 1.08, 95% CI: 1.03 to 1.12), with a 50% greater risk of SGA when comparing the highest versus lowest quartile of EDII (Table 4).

Table 5 displayed the associations of dietary anthocyanidin with the EDII. Per SD increase in the intake of anthocyanidin, the EDII decreased by 0.40 (β: −0.40, 95% CI: −0.46 to −0.34) in the multivariate model. Participants in the highest quartile of anthocyanidin intake had a 1.56−unit lower EDII (β: −1.56, 95% CI: −1.98 to −1.14) compared to those in the lowest quartile. Similar inverse associations were also observed for anthocyanidin subclasses. Our findings suggested that dietary anthocyanidin intake may mitigate the overall inflammatory potential of diet.

Table 6 showed a significant interaction between dietary anthocyanidin intake (including its subclasses) and dietary inflammatory potential on the risk of SGA (*p* interaction < 0.05). The associations between total anthocyanidin and anthocyanidin subclass intakes with SGA, stratified by EDII tertiles, were presented in Table 7. Higher intakes of total anthocyanidin (OR: 0.67, 95% CI: 0.65 to 0.68) and peonidin (OR: 0.93, 95% CI: 0.87 to 0.99) were associated with a reduced risk of SGA among participants in the highest tertile of EDII.

## 4. Discussion

To our knowledge, this was the first prospective population-based study to examine the associations between dietary intake of total anthocyanidin as well as its individual subclasses and the risk of SGA. We found that higher intake of total anthocyanidins, as well as of the cyanidin and peonidin subclasses, was inversely associated with the risk of SGA. In addition, we confirmed that a higher EDII score was associated with an increased risk of SGA, whereas higher anthocyanidin intake was inversely associated with EDII scores. The inverse association between anthocyanidin intake and SGA risk was greater among women with higher EDII scores.

In the current study, cyanidin (80.3%) and peonidin (14.6%) subclasses accounted for the majority (94.9%) of total anthocyanidin intake, with delphinidin contributing 5.1%. The primary dietary source was watermelon (98.6%), followed by sweet potato (0.6%) and grapes (0.4%). These findings aligned with previous reports identifying these foods as rich anthocyanin sources [19,35,36,37]. Notably, our findings contrasted with previous epidemiological studies identifying red wine and berries as primary anthocyanidin or anthocyanin sources in general populations [23,38,39,40]. This divergence might stem from both significantly lower red wine consumption (0.01 ± 0.27 g/day) and limited berry intake assessment via FFQ in our pregnant women. Of particular interest, we identified watermelon as a substantial contributor to anthocyanidin intake (accounting for 98.6% of total and 99.5% of cyanidin intake). This biological plausibility was supported by the verified presence of anthocyanin in watermelon rind extracts [41]. Human daily anthocyanidin intake varied considerably across diets, typically ranging from several milligrams to hundreds of milligrams. Our reported median anthocyanidin intake (28.7 mg/day) was consistent with estimates from other Asian populations, including Chinese adults (27.6 mg/day) [42] and Korean women (27.7 mg/day) [43]. Beyond this regional consistency, however, our findings highlighted substantial global geographic disparities. Anthocyanin intake varied worldwide, ranging from 12.5 mg/day in U.S. adults [44] to 24.2 mg/day in Australians [45] and 44.1 mg/day in Italians [46]. A likely explanation for the higher intake among Italians was the Mediterranean diet, which was rich in anthocyanin-containing foods such as berries and red wine. Despite being non-essential nutrients, anthocyanins had been recommended by the Chinese Nutrition Society at 50 mg/day in order to reduce oxidative stress and the risk of multiple chronic diseases [19].

To date, epidemiological evidence has found that higher consumption of anthocyanin was associated with a lower risk of several chronic diseases [47,48,49,50]. There was no evidence, to our knowledge, for a beneficial effect of anthocyanidin intake for SGA. Our findings highlighted the importance of consuming anthocyanidin for the prevention of SGA risk, which, from a public health perspective, provided support for consuming a variety of anthocyanidin-rich foods such as watermelon, sweet potatoes and grapes. This fits with our current understanding that anthocyanin could exert potential biological benefits for human health [19]. In addition, our findings confirmed that the relationship between EDII and SGA was consistent with other studies [25,51,52]. Furthermore, we found that anthocyanidin had an inverse association with EDII, and the positive EDII–SGA association was mostly apparent among women with higher EDII scores. Taken together, anthocyanidin might provide a non-pharmacological beneficial effect against SGA by reducing dietary inflammatory potential.

However, the mechanisms behind the beneficial effects of anthocyanidin at risk for SGA were poorly revealed. As well established, oxidative-antioxidative balance was crucial for implantation and embryonic development. Notably, excessive oxygen peroxidation products could induce placental degeneration and subsequent fetal growth impairment [53], while evidence further confirmed that fetal growth restriction might be associated with antioxidant deficiency [54]. Importantly, pregnancy progresses through three immunological states that align with fetal development, where an anti-inflammatory state supports fetal growth while inflammation is associated with adverse outcomes [17]. In this context, recent studies demonstrated that anthocyanins could neutralize reactive oxygen species (ROS) and activate antioxidant genes, contributing to antioxidant effects reducing inflammation [55]. Moreover, experimental and epidemiological evidence demonstrated that anthocyanins exerted anti-inflammatory effects by decreasing C-reactive protein, IL-6, TNF-α, IL-1β, and IL-8 levels [56,57,58]. At the molecular level, in vitro studies revealed that both free anthocyanin compounds (FAC) and protein-bound anthocyanin compounds (p-BAC) from purple sweet potato might exert anti-inflammatory effects via inhibition of the JNK/AP-1 signaling pathway, thereby inhibiting their downstream pro-inflammatory responses [35]. Additionally, anthocyanins extracted from a novel hybrid sweet potato peel could alleviate oxidative stress through mitigation of LPS-induced inflammation [55]. Collectively, current evidence supported that anthocyanidin might exert protective effects against SGA through comprehensive regulation of oxidative stress and inflammation.

To our knowledge, this was the first prospective population-based study to evaluate the protective role of anthocyanidin and its subclasses against SGA risk, as well as the potential modifying effect of dietary inflammatory potential. Nevertheless, several limitations should be acknowledged. First, the original research design was RCT, in which all women received multivitamins. This could potentially attenuate the influence of maternal dietary anthocyanidin on SGA, despite multivariable adjustment for multivitamins, thereby limiting the generalizability of our findings on this specific exposure. Second, anthocyanidin intake estimates likely represented conservative values due to incomplete food composition data for certain items, so we could not assume foods that were not in the reference databases did not contain anthocyanidins. Third, the current study was conducted in a rural area of China; the ethnic homogeneity of the study population might limit the generalizability of our findings, highlighting the need for confirmation in diverse ethnic groups.

## 5. Conclusions

In this prospective population-based study, we demonstrated that higher intake of anthocyanidin was protective against SGA risk. This protective effect was greater among pregnant women with higher dietary inflammatory potential. Further intervention studies in diverse populations were warranted to validate these findings.

## Figures and Tables

**Table 1:** Characteristics of the study participants across quartiles of dietary anthocyanidin intake during pregnancy in China during 2015–2019.

Baseline Characteristics	*n* = 2244	Quartiles of Anthocyanidins, mg/d				*p*
		Q1 (*n* = 561)	Q2 (*n* = 561)	Q3 (*n* = 561)	Q4 (*n* = 561)	
Anthocyanidin, range, mg/d	(0, 990.7)	(0, 13.3)	(13.3, 28.7)	(28.7, 67.8)	(67.8, 990.7)	
Anthocyanidin, median (*P*_25_, *P*_75_)	28.7 (13.3, 67.8)	6.2 (3.7, 9.9)	20.5 (16.7, 24.2)	39.4 (34.3, 52.7)	131.8 (103.1, 198.6)	
Season of birth						<0.001
Spring (March-May), *n* (%)	467 (20.8)	114 (25.7)	144 (25.7)	77 (13.7)	48 (8.56)	
Summer (June-August), *n* (%)	509 (22.7)	114 (20.3)	114 (20.3)	137 (24.4)	133 (23.7)	
Autumn (September-November), *n* (%)	661 (29.5)	149 (26.6)	149 (26.6)	194 (34.6)	223 (39.8)	
Winter (December-February), *n* (%)	607 (27.1)	154 (27.5)	154 (27.5)	153 (27.3)	157 (28.0)	
Multivitamin supplement, *n* (%)						0.931
Folic acid	814 (36.3)	201 (35.8)	214 (38.2)	206 (36.7)	193 (34.4)	
Folic acid plus iron	704 (31.4)	176 (31.4)	158 (28.2)	177 (31.6)	193 (34.4)	
Folic acid plus B-complex vitamins	726 (32.4)	184 (32.8)	189 (33.7)	178 (31.7)	175 (31.2)	
Maternal age (years), mean ± SD	25.8 ± 4.1	26.0 ± 4.4	25.8 ± 4.2	25.5 ± 3.6	25.9 ± 4.1	0.193
Parity, *n* (%)						<0.001
Nulliparous	1095 (48.8)	279 (49.7)	296 (52.8)	265 (47.2)	255 (45.5)	
Multiparous	1149 (51.2)	282 (50.3)	365 (47.2)	296 (52.8)	306 (54.6)	
Socioeconomic status ^a^, *n* (%)						0.800
Lower	747 (33.3)	190 (33.9)	178 (31.7)	183 (32.6)	196 (34.9)	
Medium	747 (33.3)	195 (34.8)	184 (32.8)	198 (35.3)	170 (30.3)	
Upper	750 (33.4)	176 (31.4)	199 (35.5)	180 (32.1)	195 (34.8)	
Body mass index (kg/m^2^), mean ± SD	21.4 ± 2.7	21.4 ± 2.8	21.5 ± 2.7	21.5 ± 2.7	21.4 ± 2.5	0.631
Passive smoking ^b^, *n* (%)	248 (11.1)	67 (11.9)	61 (10.9)	54 (9.6)	66 (11.8)	0.354
Pregnancy complication ^c^, *n* (%)	70 (3.1)	29 (5.2)	17 (3.0)	7 (1.3)	17 (3.0)	<0.001
Birthweight, gram, mean ± SD	3240.2 ± 430.3	3234.8 ± 428.5	3219.0 ± 435.6	3251.9 ± 427.5	3255.1 ± 429.8	0.114
Gestational age, weeks, mean ± SD	39.7 ± 1.2	39.7 ± 1.2	39.6 ± 1.3	39.7 ± 1.2	39.7 ± 1.3	0.210
Small-for-gestational-age, *n* (%)	234 (10.4)	62 (11.1)	57 (10.2)	64 (11.4)	51 (9.1)	0.033
EDII, mean ± SD	−0.002 ± 1.805	0.63 ± 1.77	0.21 ± 1.74	−0.11 ± 1.74	0.75 ± 1.69	<0.001

Q, quartile. EDII, energy-adjusted dietary inflammatory index. Values are mean ± standard deviations or *n* (%). Tests of significance are adjusted for clustering by county using a generalized estimating equation. ^a^ Socioeconomic status was assessed using principal component analysis to derive a composite score from five indicators: family wealth index, maternal and paternal education levels, and maternal and paternal occupations. Socioeconomic status was then categorized into lower, medium, and higher groups based on tertiles of the composite score. ^b^ Passive smoking was defined as exposure to secondhand smoke for more than 15 min daily during pregnancy. ^c^ Pregnancy complications included pregnancy-induced hypertension syndrome and gestational diabetes mellitus.

**Table 2:** Correlation of food parameters with anthocyanidin intake and their distribution across anthocyanidin intake quartiles.

Food Parameters	Correlation with Anthocyanidin		Quartiles of Anthocyanidin, mg/d			
	Pearson’s *r*	*p*	Q1	Q2	Q3	Q4
Energy (kcal/d)	0.24	<0.001	2041.2 ± 678.8	2231.1 ± 675.6	2373.4 ± 678.6	2534.8 ± 719.3
Carbohydrate (g/d)	0.22	<0.001	322.1 ± 126.3	355.3 ± 125.6	383.3 ± 124	407.1 ± 132.5
Protein (g/d)	0.18	<0.001	58.1 ± 22.1	64.6 ± 22.3	67.7 ± 22.7	72.2 ± 24.1
Total fat (g/d)	0.18	<0.001	64.4 ± 21.9	68.7 ± 22.1	71.2 ± 24.7	76.9 ± 24.6
Saturated fat (g/d)	0.13	<0.001	13.8 ± 6.6	15.2 ± 6.7	15.7 ± 7.0	16.9 ± 7.5
MUFA (g/d)	0.14	<0.001	27.6 ± 7.0	29.3 ± 7.0	30.1 ± 8.0	31.3 ± 8.0
PUFA (g/d)	0.17	<0.001	17.6 ± 10.1	18.8 ± 10.4	19.8 ± 12.3	22.6 ± 11.9
*n*-3 fatty acids (g/d)	0.15	<0.001	5.2 ± 3.9	5.4 ± 4.0	5.7 ± 4.8	6.7 ± 4.7
*n*-6 fatty acids (g/d)	0.17	<0.001	17.7 ± 10.1	18.8 ± 10.5	19.9 ± 12.4	22.7 ± 12.0
Cholesterol (mg/d)	0.04	0.064	197.8 ± 144.9	218.2 ± 142.3	217.7 ± 143.9	223.7 ± 152.9
Fiber (g/d)	0.31	<0.001	8.6 ± 4.8	10.1 ± 5.0	11.1 ± 5.4	13.6 ± 6.3
Vitamin A (μg/d, RAE)	0.15	<0.001	294.6 ± 259.5	336.8 ± 207.6	359.0 ± 219.2	435.3 ± 371.7
β-Carotene (μg/d)	0.27	<0.001	1613.5 ± 1166.4	1989.1 ± 1309.2	2246.2 ± 1431	2755.7 ± 1481.8
Thiamin (mg/d)	0.29	<0.001	0.7 ± 0.3	0.8 ± 0.3	0.9 ± 0.3	1.0 ± 0.3
Riboflavin (mg/d)	0.25	<0.001	0.9 ± 0.6	1.0 ± 0.5	1.1 ± 0.5	1.3 ± 0.6
Niacin (mg/d)	0.29	<0.001	11.3 ± 4.2	12.9 ± 4.3	13.8 ± 4.9	15.5 ± 5.3
Vitamin B6 (mg/d)	0.35	<0.001	1.1 ± 0.5	1.2 ± 0.5	1.4 ± 0.6	1.7 ± 0.6
Vitamin B12 (μg/d)	0.004	0.842	4.0 ± 4.5	4.5 ± 4.3	4.8 ± 4.0	4.7 ± 5.0
Folic acid (μg/d)	0.26	<0.001	234.4 ± 119.6	270.8 ± 125.3	295.2 ± 142.4	351.5 ± 152.8
Vitamin C (mg/d)	0.34	<0.001	89.4 ± 56.7	103.3 ± 54.3	116.6 ± 58.4	148.7 ± 67
Vitamin D (μg/d)	0.08	<0.001	1.8 ± 1.5	2.0 ± 1.5	1.9 ± 1.4	2.2 ± 1.5
Vitamin E (mg/d)	0.18	<0.001	35.4 ± 18.8	37.6 ± 19.2	39.7 ± 23	45.1 ± 22.7
Mg (mg/d)	0.23	<0.001	369 ± 157.9	411.2 ± 159.6	442.6 ± 156.2	488.1 ± 168.1
Fe (mg/d)	0.25	<0.001	16.7 ± 6.3	18.7 ± 6.3	20.2 ± 7.6	22.7 ± 8.4
Zn (mg/d)	0.21	<0.001	5.8 ± 2.4	6.6 ± 2.4	6.9 ± 2.8	7.7 ± 3
Se (μg/d)	0.14	<0.001	26.9 ± 12.3	29.9 ± 12.9	32.1 ± 14.7	33.7 ± 15.2
Isoflavones (mg/d)	0.12	<0.001	0.8 ± 0.5	0.9 ± 0.5	0.9 ± 0.9	1.0 ± 0.8
Caffeine (g/d)	0.04	0.070	1.0 ± 7.0	1.3 ± 8.0	1.2 ± 4.3	1.3 ± 4.7
Alcohol (g/d)	0.02	0.449	0.01 ± 0.10	0.01 ± 0.08	0.003 ± 0.047	0.02 ± 0.34
Green/black tea (g/d)	0.003	0.903	3.5 ± 34.7	3.9 ± 39.1	3.7 ± 20.5	3.3 ± 20.5
Onion (g/d)	0.14	<0.001	4.0 ± 9.6	5.1 ± 8.4	7.2 ± 12.2	8.9 ± 16.7
Garlic (g/d)	0.02	0.336	3.7 ± 10.7	3.3 ± 5.5	4.8 ± 12.5	4.1 ± 5.7

Q, quartile. Values are means ± standard deviations.

**Table 3:** Explained interindividual variance in the anthocyanidins by food sources among pregnant women in China during 2015–2019.

Food Source	*R* ^2^	Model *R*^2^
Anthocyanidins		
Watermelon	0.986	0.986
Sweet potato	0.006	0.991
Grapes	0.004	0.995
Plum/apricot	0.003	0.999
Eggplant	0.001	1.000
Delphinidin		
Eggplant	0.495	0.495
Sweet potato	0.307	0.802
Grapes	0.166	0.968
Persimmon	0.031	0.999
Banana	0.001	1.000
Cyanidin		
Watermelon	0.995	0.995
Plum/apricot	0.003	0.999
Eggplant	0.001	1.000
Peonidin		
Sweet potato	0.584	0.584
Grapes	0.417	1.000

Forward linear regression was used to calculate the *R*^2^ and model *R*^2^. Food groups that explain 0.1% of the interindividual variation are shown.

**Table 4:** Associations of anthocyanidin and dietary inflammatory index with the risk of small-for-gestational-age in China during 2015–2019.

	Continuous	Quartile of Anthocyanidin or EDII			
		Q1	Q2	Q3	Q4
Total anthocyanidin					
Cases/subjects	234/2244	62/561	57/561	64/561	51/561
Median (25th, 75th), mg/d	28.67 (13.29, 67.78)	6.21 (3.68, 9.86)	20.45 (16.65, 24.18)	39.43 (34.32, 52.73)	131.82 (103.12, 198.56)
Model 1, OR (95%CI)	0.96 (0.95, 0.97)	referent	0.91 (0.69, 1.19)	1.04 (0.88, 1.23)	0.80 (0.66, 0.97)
Model 2, OR (95%CI)	0.96 (0.95, 0.97)	referent	0.91 (0.70, 1.18)	1.04 (0.89, 1.21)	0.81 (0.66, 0.98)
Individual anthocyanidin					
Delphinidin					
Cases/subjects	234/2244	60/561	58/561	58/561	58/561
Median (25th, 75th), mg/d	0.83 (0.44, 1.69)	0.24 (0.15, 0.35)	0.60 (0.51, 0.71)	1.14 (0.99, 1.35)	2.75 (2.26, 3.62)
Model 1, OR (95%CI)	1.02 (0.98, 1.06)	referent	0.96 (0.85, 1.09)	0.96 (0.84, 1.10)	0.96 (0.88, 1.06)
Model 2, OR (95%CI)	1.03 (0.99, 1.07)	referent	0.97 (0.85, 1.10)	0.96 (0.84, 1.11)	0.99 (0.93, 1.07)
Cyanidin					
Cases/subjects	234/2244	60/561	57/561	66/561	51/561
Median (25th, 75th), mg/d	23.56 (8.40, 62.56)	3.53 (1.96, 5.40)	15.30 (12.29, 19.99)	33.89 (28.93, 45.81)	125.08 (95.01, 188.15)
Model 1, OR (95%CI)	0.96 (0.95, 0.97)	referent	0.94 (0.78, 1.14)	1.11 (0.91, 1.36)	0.84 (0.72, 0.97)
Model 2, OR (95%CI)	0.96 (0.95, 0.97)	referent	0.93 (0.77, 1.12)	1.10 (0.97, 1.34)	0.83 (0.71, 0.97)
Peonidin					
Cases/subjects	234/2244	65/554	55/566	56/571	58/553
Median (25th, 75th), mg/d	2.20 (0.88, 4.64)	0.27 (0.00, 0.53)	1.51 (1.14, 1.85)	3.06 (2.64, 3.65)	9.85 (6.75, 13.40)
Model 1, OR (95%CI)	0.96 (0.94, 0.97)	referent	0.81 (0.75, 0.87)	0.81 (0.69, 0.95)	0.88 (0.80, 0.97)
Model 2, OR (95%CI)	0.96 (0.96, 0.97)	referent	0.81 (0.74, 0.89)	0.80 (0.67, 0.95)	0.89 (0.79, 1.00)
EDII					
Cases/subjects	234/2244	42/561	68/561	63/561	61/561
Means ± SD	−0.002 ± 1.805	0.63 ± 1.77	0.21 ± 1.74	−0.11 ± 1.74	0.75 ± 1.69
Model 1, OR (95%CI)	1.08 (1.03, 1.12)	referent	1.70 (1.43, 2.03)	1.56 (1.22, 2.00)	1.51 (1.11, 2.04)
Model 2, OR (95%CI)	1.08 (1.03, 1.12)	referent	1.73 (1.43, 2.10)	1.56 (1.19, 2.05)	1.50 (1.08, 2.08)

EDII, energy-adjusted dietary inflammatory index; Q, quartile; SD, standard deviations. Analysis using generalized estimating equations. Odds ratios (ORs) with 95% confidence intervals (95% CIs) were estimated for a 1-SD increment in continuous dietary anthocyanidins. Model 1 adjusted for clustering by country using generalized estimating equations. Model 2 adjusted for multivitamin supplement (folic acid, folic acid plus iron, and folic acid plus B-complex vitamins), intake of total energy, birth season (spring, summer, autumn, and winter), maternal age (continuous), nulliparity status (nulliparity or multiparity), socioeconomic situation (lower, medium, and upper), passive smoking (yes or no), maternal body mass index at enrollment (continuous), and pregnancy complication (hypertension or gestational diabetes). For the effect of peonidin in model 2, the OR (95%CI) was 0.886 (0.786, 0.997).

**Table 5:** Associations of dietary anthocyanidin with the dietary inflammatory index in China during 2015–2019.

	Continuous	Quartile of Anthocyanidin, mg/d			
		Q1	Q2	Q3	Q4
Total anthocyanidin					
Median (25th, 75th), mg/d	28.67 (13.29, 67.78)	6.21 (3.68, 9.86)	20.45 (16.65, 24.18)	39.43 (34.32, 52.73)	131.82 (103.12, 198.56)
Model 1, β (95%CI)	−0.36 (−0.41, −0.33)	referent	−0.42 (−0.58, −0.26)	−0.74 (−1.03, −0.45)	−1.39 (−1.76, −1.02)
Model 2, β (95%CI)	−0.40 (−0.46, −0.34)	referent	−0.48 (−0.62, −0.34)	−0.88 (−1.17, −0.59)	−1.56 (−1.98, −1.14)
Individual anthocyanidin					
Delphinidin					
Median (25th, 75th), mg/d	0.44 (0.83, 1.69)	0.24 (0.15, 0.35)	0.60 (0.51, 0.71)	1.14 (0.99, 1.35)	2.75 (2.26, 3.62)
Model 1, β (95%CI)	−0.65 (−0.70, −0.60)	referent	−0.56 (−0.65, −0.47)	−1.42 (−1.54, −1.30)	−1.94 (−2.04, −1.84)
Model 2, β (95%CI)	−0.74 (−0.78, −0.69)	referent	−0.66 (−0.78, −0.55)	−1.56 (−1.72, −1.41)	−2.20 (−2.35, −2.05)
Cyanidin					
Median (25th, 75th), mg/d	8.40 (23.56, 62.56)	3.53 (1.96, 5.40)	15.30 (12.29, 19.99)	33.89 (28.93, 45.81)	125.08 (95.01, 188.15)
Model 1, β (95%CI)	−0.33 (−0.37, −0.29)	referent	−0.01 (−0.26, 0.24)	−0.44 (−0.66, −0.23)	−1.07 (−1.56, −0.59)
Model 2, β (95%CI)	−0.36 (−0.41, −0.30)	referent	−0.07 (−0.27, 0.14)	−0.55 (−0.72, −0.39)	−1.21 (−1.70, −0.71)
Peonidin					
Median (25th, 75th), mg/d	0.88 (2.20, 4.64)	0.27 (0.00, 0.53)	1.51 (1.14, 1.85)	3.06 (2.64, 3.65)	9.85 (6.75, 13.40)
Model 1, β (95%CI)	−0.53 (−0.57, −0.51)	referent	−0.36 (−0.53, −0.18)	−1.06 (−1.13, −0.98)	−1.67 (−1.71, −1.63)
Model 2, β (95%CI)	−0.58 (−0.61, −0.54)	referent	−0.37 (−0.57, −0.18)	−1.12 (−1.21, −1.02)	−1.78 (−1.87, −1.70)

Q, quartile. Analysis using generalized estimating equations. Model 1 adjusted for clustering by country using generalized estimating equations. Model 2 adjusted for multivitamin supplement (folic acid, folic acid plus iron, and folic acid plus B-complex vitamins), intake of total energy, birth season (spring, summer, autumn, and winter), maternal age (continuous), nulliparity status (nulliparity or multiparity), socioeconomic situation (lower, medium, and upper), passive smoking (yes or no), maternal body mass index at enrollment (continuous), and pregnancy complication (hypertension or gestational diabetes).

**Table 6:** Interaction effect of dietary anthocyanidin and dietary inflammatory potential on small-for-gestational-age in China during 2015–2019.

	OR (95%CI)	*p*
EDII	1.08 (1.03, 1.12)	<0.001
Total anthocyanidin		
Anthocyanidin	0.97 (0.97, 0.98)	<0.001
EDII	1.10 (1.04, 1.17)	0.002
Anthocyanidin × EDII	0.96 (0.94, 0.98)	<0.001
Individual anthocyanidin		
Delphinidin	1.05 (1.02, 1.09)	0.005
EDII	1.15 (1.12, 1.19)	<0.001
Delphinidin × EDII	0.95 (0.93, 0.96)	<0.001
Cyanidin	0.97 (0.97, 0.98)	<0.001
EDII	1.10 (1.03, 1.17)	0.004
Cyanidin × EDII	0.97 (0.94, 0.99)	0.010
Peonidin	0.96 (0.92, 1.01)	0.093
EDII	1.11 (1.09, 1.14)	<0.001
Peonidin × EDII	0.95 (0.92, 0.98)	0.004

EDII, energy-adjusted dietary inflammatory index. Odds ratios (ORs) with 95% confidence intervals (95% CIs) were estimated using generalized estimating equations adjusted for clustering, multivitamin supplements (folic acid, folic acid plus iron, and folic acid plus B-complex vitamins), intake of total energy, birth season (spring, summer, autumn, and winter), maternal age (continuous), nulliparity status (nulliparity or multiparity), socioeconomic situation (lower, medium, and upper), passive smoking (yes or no), maternal body mass index at enrollment (continuous), and pregnancy complications (hypertension or gestational diabetes).

**Table 7:** Stratified analysis of the association between dietary anthocyanidin and small-for-gestational-age by tertiles of energy-adjusted dietary inflammatory index in China during 2015–2019.

	Tertiles of Energy-Adjusted Dietary Inflammatory Index		
	T_1_	T_2_	T_3_
Total anthocyanidin			
Median (25th, 75th), mg/d	41.24 (18.92, 112.50)	30.66 (13.85, 67.83)	21.12 (8.40, 36.39)
OR (95%CI)	1.08 (0.96, 1.21)	1.04 (0.89, 1.22)	0.67 (0.65, 0.68)
Individual anthocyanidin			
Delphinidin			
Median (25th, 75th), mg/d	1.35 (0.77, 2.61)	0.84 (0.49, 1.52)	0.49 (0.25, 0.91)
OR (95%CI)	1.10 (1.00, 1.45)	1.02 (0.88, 1.18)	0.88 (0.69, 1.10)
Cyanidin			
Median (25th, 75th), mg/d	32.95 (13.23, 107.22)	25.10 (8.96, 63.72)	17.03 (5.47, 33.31)
OR (95%CI)	1.10 (0.94, 1.29)	0.95 (0.81, 1.11)	0.91 (0.82, 1.02)
Peonidin			
Median (25th, 75th), mg/d	3.45 (1.57, 7.74)	2.28 (1.05, 4.64)	1.23 (0.53, 2.55)
OR (95%CI)	1.06 (0.97, 1.15)	0.97 (0.90, 1.06)	0.93 (0.87, 0.99)

T, tertiles. Odds ratios (ORs) with 95% confidence intervals (95% CIs) were estimated using generalized estimating equation adjusted for clustering, multivitamin supplements (folic acid, folic acid plus iron, and folic acid plus B-complex vitamins), intake of total energy, birth season (spring, summer, autumn, and winter), maternal age (continuous), nulliparity status (nulliparity or multiparity), socioeconomic situation (lower, medium, and upper), passive smoking (yes or no), maternal body mass index at enrollment (continuous), and pregnancy complications (hypertension or gestational diabetes).

## Data Availability

The original contributions presented in the study are included in the article Further inquiries can be directed to the corresponding author S.D. (tjdshn@xjtu.edu.cn).

## Supplementary Materials

The following supporting information can be downloaded at: https://www.mdpi.com/article/10.3390/nu17203187/s1, Figure S1: Study participant flow chart in China.
