# Supplementary figures and images for: Maternal Dietary Anthocyanidin, Dietary Inflammatory Potential, and Risk of Small-for-Gestational-Age in China
Source: Nutrients. 2025 Oct 10;17(20):3187. doi: 10.3390/nu17203187 (PMC12567153; doi:10.3390/nu17203187)

**Supplementary Figure S1.** Study participant flow chart in China.

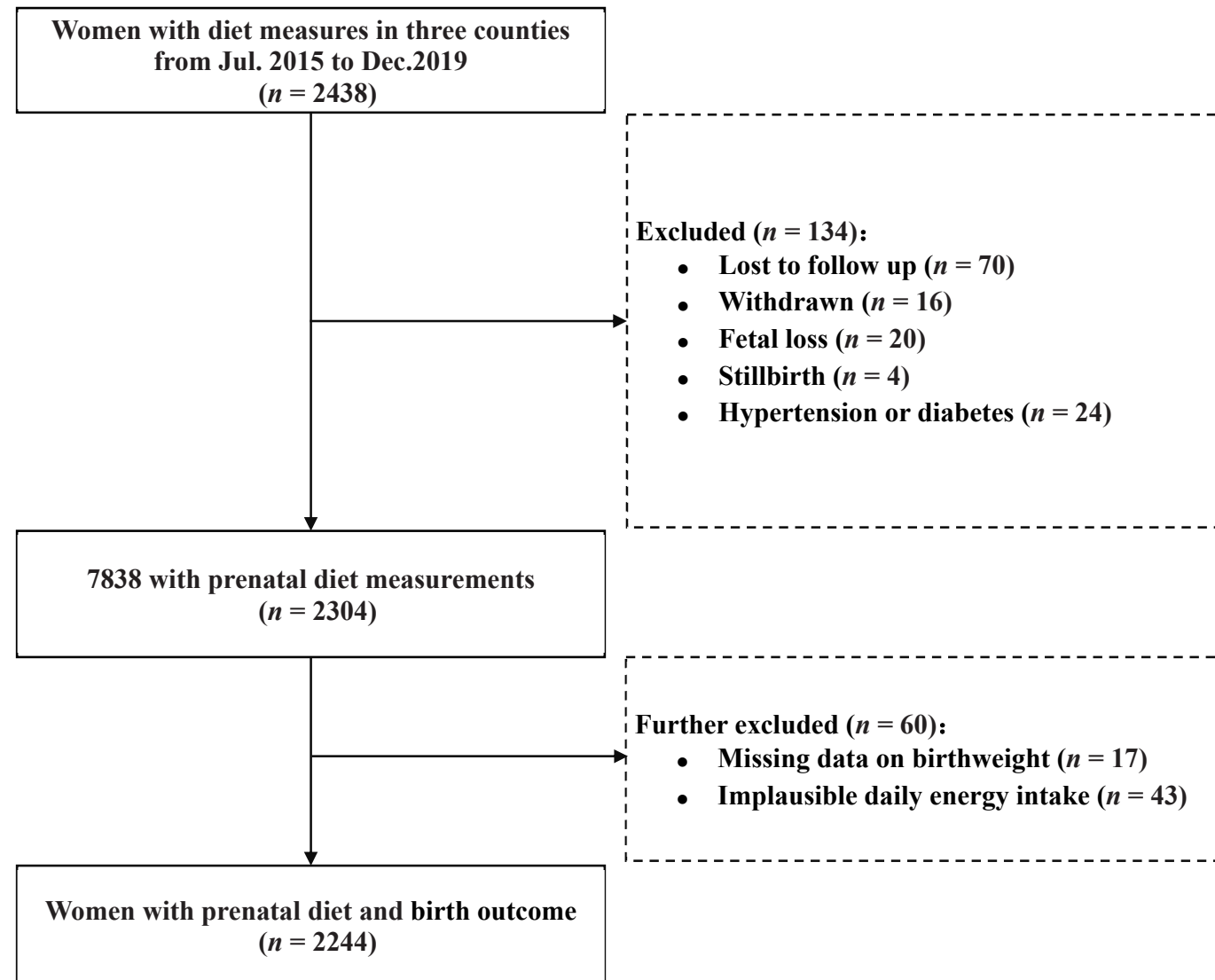

Supplement: Supplementary file 1 [file nutrients-17-03187-s001.zip › nutrients-3889452-supplementary.pdf]
